# Three-Dimensional Tumor Models to Study Cancer Stemness-Mediated Drug Resistance

**DOI:** 10.1007/s12195-024-00798-y

**Published:** 2024-02-21

**Authors:** Astha Lamichhane, Hossein Tavana

**Affiliations:** Department of Biomedical Engineering, The University of Akron, Akron, OH 44325 USA

**Keywords:** Drug resistance, Cancer stem cells, Spheroids, Organoids, Microfluidic devices, Tumor microenvironment, 3D tumor models, Stemness

## Abstract

Solid tumors often contain genetically different populations of cancer cells, stromal cells, various structural and soluble proteins, and other soluble signaling molecules. The American Cancer society estimated 1,958,310 new cancer cases and 609,820 cancer deaths in the United States in 2023. A major barrier against successful treatment of cancer patients is drug resistance. Gain of stem cell-like states by cancer cells under drug pressure or due to interactions with the tumor microenvironment is a major mechanism that renders therapies ineffective. Identifying approaches to target cancer stem cells is expected to improve treatment outcomes for patients. Most of our understanding of drug resistance and the role of cancer stemness is from monolayer cell cultures. Recent advances in cell culture technologies have enabled developing sophisticated three-dimensional tumor models that facilitate mechanistic studies of cancer drug resistance. This review summarizes the role of cancer stemness in drug resistance and highlights the various tumor models that are used to discover the underlying mechanisms and test potentially novel therapeutics.

## Introduction

Cancer is the second leading cause of mortality with about 21% of all deaths in developed countries [1]. The most lethal cancers are breast, cervical, lung, thyroid, and colorectal cancers in women, and prostate, lung, colorectal, liver, and stomach cancers in men [2]. Surgery, chemotherapy, hormone therapy, gene therapy, immunotherapy, radiation therapy, laser therapy, combination therapy, and targeted therapy are the main treatments available, with chemotherapy being the most common across different cancers [3, 4]. Despite the progress made in cancer treatments, therapy resistance remains a major clinical problem and hampers the durability of the treatments. Although in many cases cancer cells initially respond to the treatments, they often display resistance over time and eventually lead to treatment failure. Nevertheless, advances in high throughput screening of cancer drug candidates and genomic profiling of tumors to facilitate precision cancer medicine have created opportunities to understand specific mechanisms of therapy resistance and develop more effective and durable treatment strategies in several cancers. Between 1991 and 2017, significant improvements in precision medicine and targeted therapies led to a notable 29% reduction in cancer mortality rates in the United State [5]. However, despite these advancements, cancer remains a major clinical problem, emphasizing the need for mechanistic understanding of drug resistance to develop treatment strategies that improve outcomes for patients.

## Cancer Drug Resistance

**Fig. 1:**
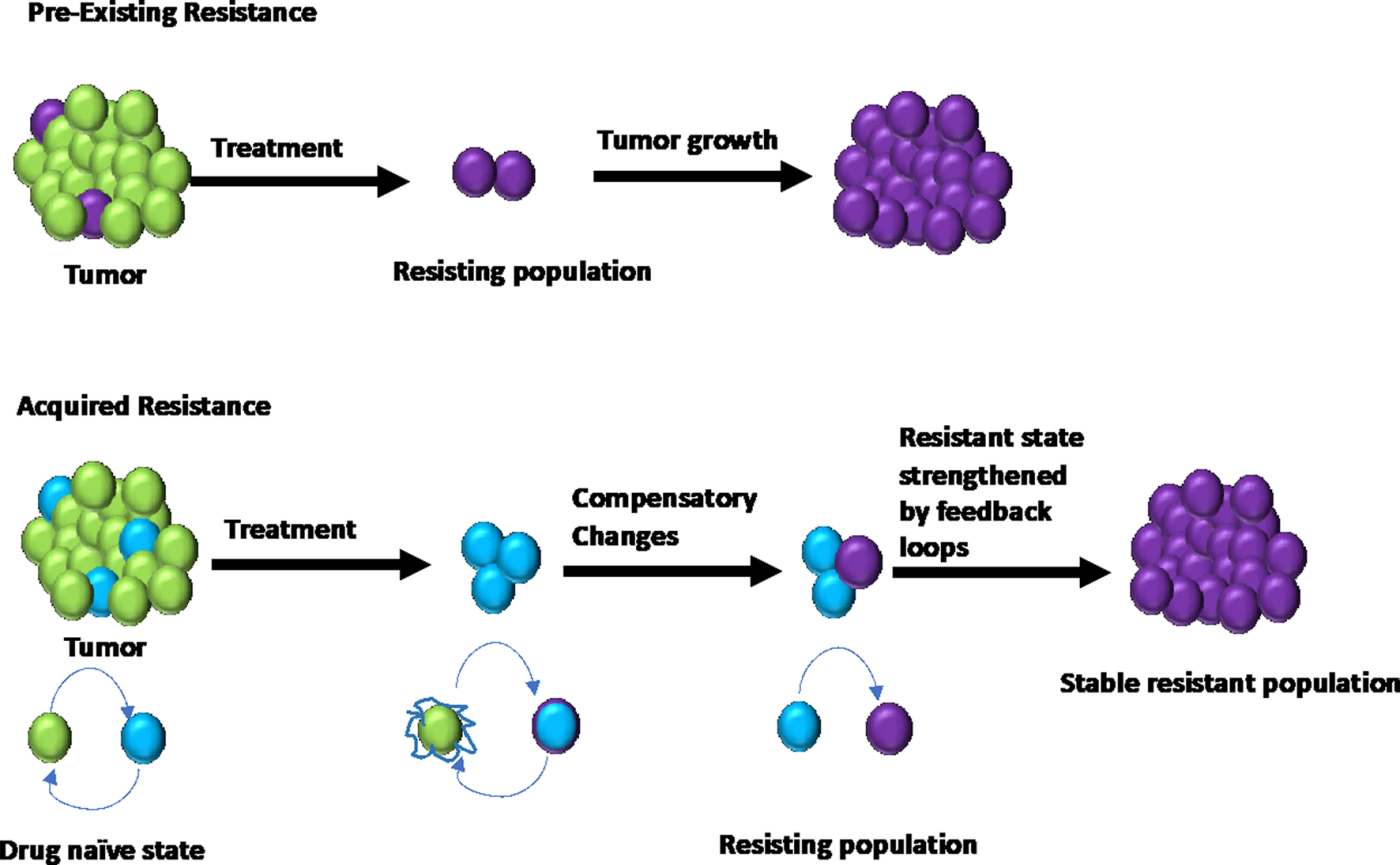
Mechanisms of cancer drug resistance. Green cells represent the bulk of tumor, purple cells represent the pre-existing resistant population, and blue cells represent the population that undergo changes in response to the drug and develop acquired resistance. Adapted from Bell, C.C. et al.; Br. J. Cancer 122 (2020) (© 2019, Charles C. Bell et al, under exclusive license to Cancer Research UK)

Resistance to drugs results when cancer cells become tolerant to pharmaceuticals used to treat cancer. Drug resistance and subsequent disease progression to metastasis is responsible for over 90% of mortality of cancer patients [6]. Drug resistance is classified into two broad categories – intrinsic and acquired resistance [7, 8]. Intrinsic resistance is due to the preexisting heterogeneity of cancer cells in the bulk of a tumor and exists before starting the treatment. The determinants of intrinsic resistance are inherent genetic mutations in tumor cells [9], heterogeneity in the makeup of the population of tumor cells [10], differences in activities of oncogenic signaling pathways in cancer cells [11], and other pharmacological factors than cancer patients may be exposed to. Thus, the stable resistant state can result from pre-existing resistance (Fig. 1).

Acquired resistance occurs by selecting for cells that gradually develop resistance to treatment pressure to form a stably resistant tumor. While cytotoxic chemotherapeutics primarily target actively proliferating cells, the use of modern targeted therapies following analysis of tumor biopsies aims to target main molecular drivers of tumorigenesis. Therefore, non-proliferative cells in tumors, slow-cycling cells due to nutrients limitations, quiescent stem-like cells, and cells not represented as primary drivers of the tumor growth in biopsy analysis often escape the therapies. And under drug pressure, these cells can transition to a new, stable resistant state and promote tumor relapse (Fig. 1) [12, 13]. Acquired resistance is characterized by the reduced treatment efficacy due to the drug-resistant cell populations. The mechanisms underlying acquired resistance include, but are not limited to, mutations of drug targets, activation of new proto-oncogenes, signaling from the tumor microenvironment, epigenetic changes by methylation, acetylation, and microRNA (miRNA) expression that lead to alterations in upstream or downstream regulators, alterations in the cell cycle and its checkpoints, and altered DNA repair [14, 15]. Intrinsic and acquired resistance can occur concurrently in tumors and understanding the underlying complex mechanisms is critical for developing more effective cancer treatments that address both pathways to resistance.

## Cancer Stem Cells

Cancer stem cells (CSCs) are generally defined as a small population of tumor cells that have the capacity to self-renew and differentiate into heterogenous cells comprising the tumor. CSCs possess a high tumorigenic capacity, remain in quiescent state under treatment pressure and show resistance to different cancer therapies [16–18]. Commonly used chemotherapeutic agents mainly eradicate the bulk of proliferating tumor cells and spare CSCs, which can lead to cancer growth and relapse [19, 20]. The first evidence for the presence of CSCs in solid cancers emerged from identifying CD44^+^/CD24^-/low^ lineage cells in immunocompromised mice with transplanted human breast cancer cells [21]. CSCs have been identified in several other solid cancers including melanoma [22], brain [23], lung [24], liver [25], pancreas [26], colon [27], and breast and ovarian cancers [28].

**Fig. 2:**
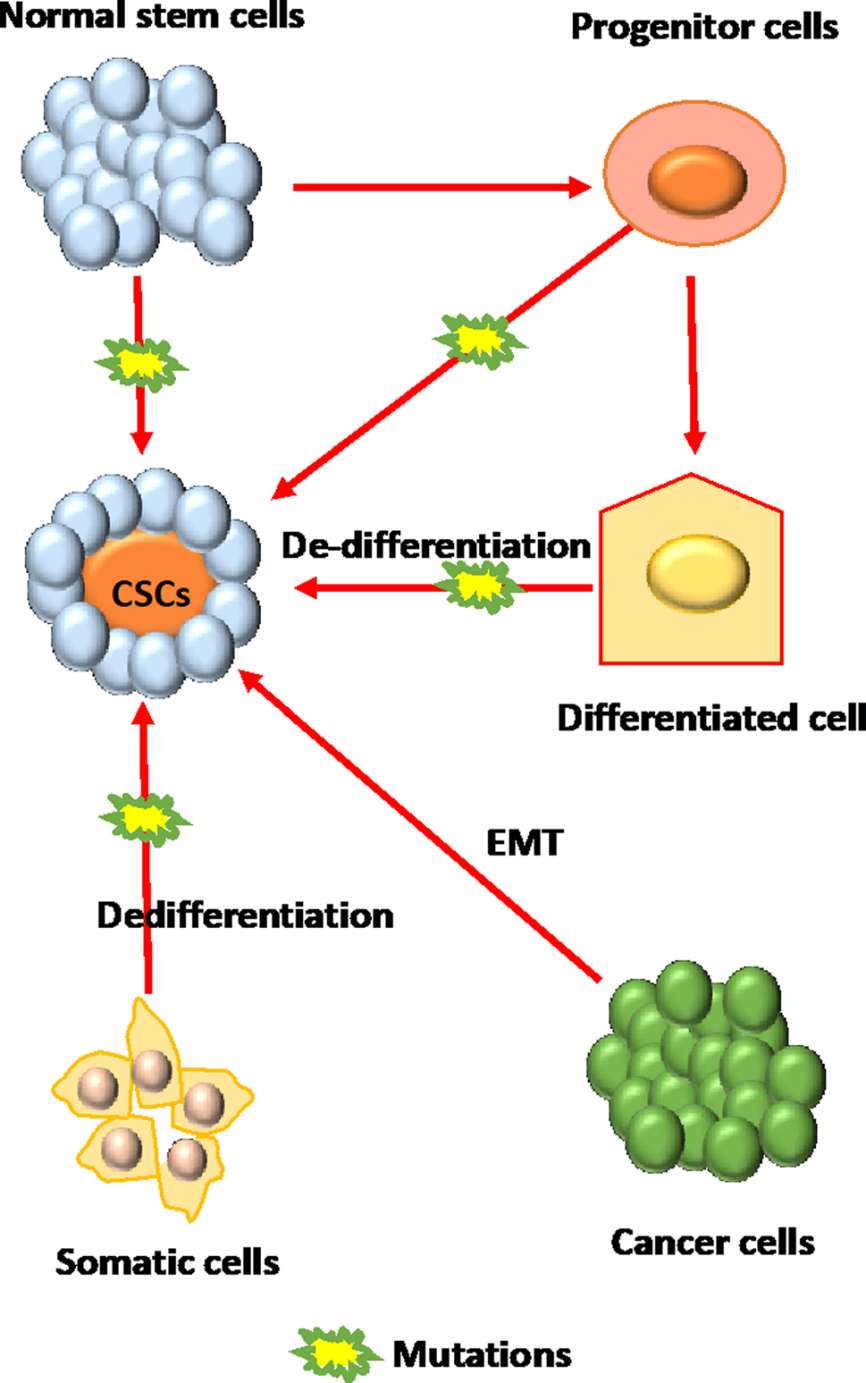
Cancer stem cells (CSCs) can develop from normal stem cells and progenitor cells through mutations, from dedifferentiated somatic cells, or from cancer cells undergoing EMT. Adapted from Khan, A.Q. et al.; Cells 8 (2019) (© 2019 by Khan A Q et al.)

Different hypotheses suggest that depending on the tumor type, CSCs might be derived from adult stem cells, mutated adult progenitor cells, dedifferentiated somatic cells or cancer cells with plasticity to obtain stem-like properties through dedifferentiation (Fig. 2) [29–32]. The expression levels of several cell surface markers, including CD133, CD24, CD44, and EpCAM are commonly used to identify CSCs [21, 33]. Additionally, aldehyde dehydrogenase 1 (ALDH1) is used to characterize CSCs in breast, colon, liver, lung, and pancreatic cancers [34, 35]. Elucidating the diversity of CSCs and identifying their markers across different tumor types will help develop new targeted therapies to eliminate CSCs, improve therapy outcomes, and prevent relapse. However, the heterogeneity of CSCs and the specificity of their biomarker to each tumor type or even tumor subtype are significant challenges to overcome.

## Drug Resistance Mechanisms of CSCs

**Fig. 3:**
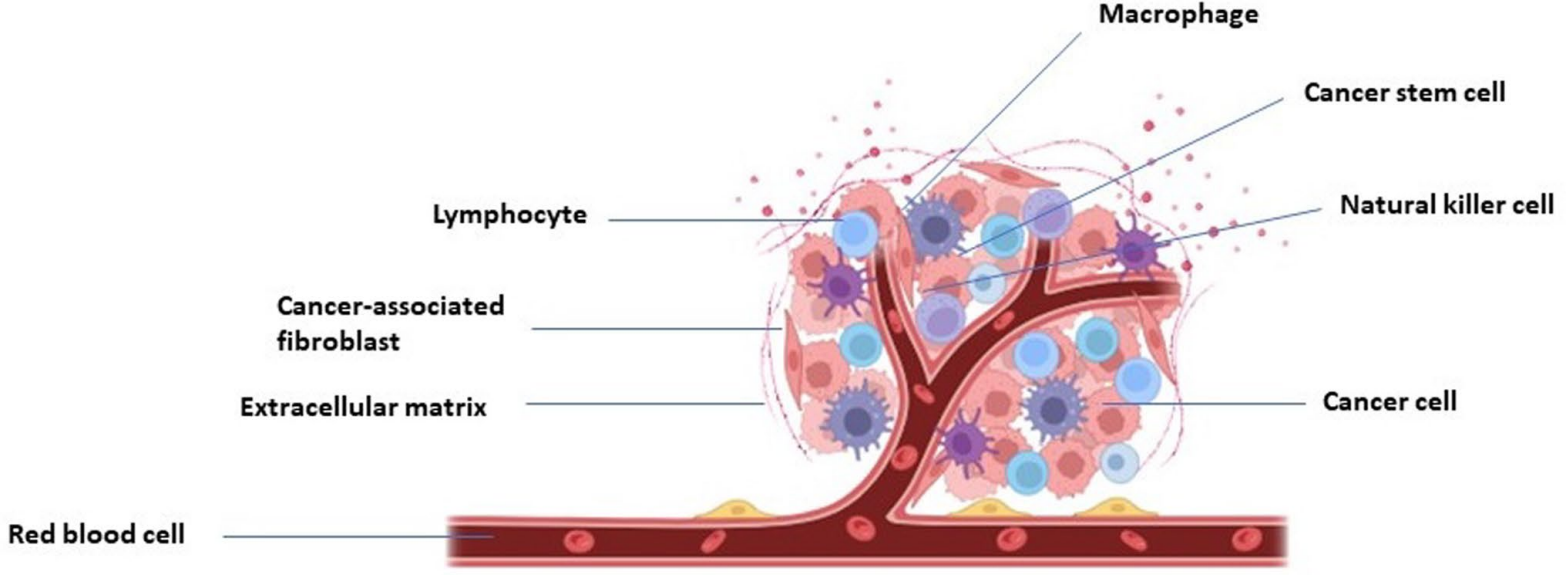
Representation of tumor microenvironment consisting of macrophages, CSCs, natural killer cells, cancer cells, red blood cells, ECM, cancer-associated fibroblasts (CAFs), and lymphocytes. Adapted from ‘Tumor microenviroment’ by BioRender.com.

Different mechanisms involved in drug resistance of CSCs include cell-intrinsic changes such as epithelial-to-mesenchymal transition (EMT) or switch from a proliferative state to a dormant state, and cell-extrinsic factors such as hypoxia or signaling from the tumor microenvironment. For example, EMT markers and stem cell markers were co-expressed in circulating tumor cells from patients with metastasis [36], and EMT induction or activation of EMT transcription factors conferred stem-like properties in cancer cells [37]. In bladder cancer, quiescent CSCs led to an unexpected cell division to repopulate residual tumors between chemotherapy cycles [38]. Tumor microenvironment (Fig. 3) contains various stromal cells and soluble signaling factors that can stimulate signaling pathways in cancer cells, such as Notch [39] and Wnt [40], that are essential pathways for CSCs. Cell-extrinsic factors from the tumor microenvironment can also induce EMT and dormancy in cancer cells. For example, interactions of bone marrow mesenchymal stem cells (MSCs) with metastasized ER^+^ (estrogen receptor-positive) breast cancer cells in bone marrow led to metabolic shift and dormancy of cancer cells and resistance to endocrine therapies [41]. In addition, tumor hypoxia was shown to promote CSCs and EMT phenotypes [42], e.g., via an increased expression of VEGF, IL-6, and CSC signature genes such as Nanog and Oct4 [43]. In prostate cancer, hypoxia elevated the expression of stemness gene markers Nanog, Oct3/4, and SOX2 [44]. Low extracellular pH is a signature of hypoxic regions in solid tumors. Tumor acidosis shifts HIF1α-driven glycolytic metabolism of cancer cells toward a metabolism that relies on glutamine and lipids as preferred sources of energy, suppresses immunosurveillance, and promotes chemoresistance [45]. Using drugs that interfere with H^+^ or bicarbonate transporters or exploiting pH-sensitive drug delivery systems can potentially be leveraged as therapeutic strategies in solid tumors.

**Fig. 4:**
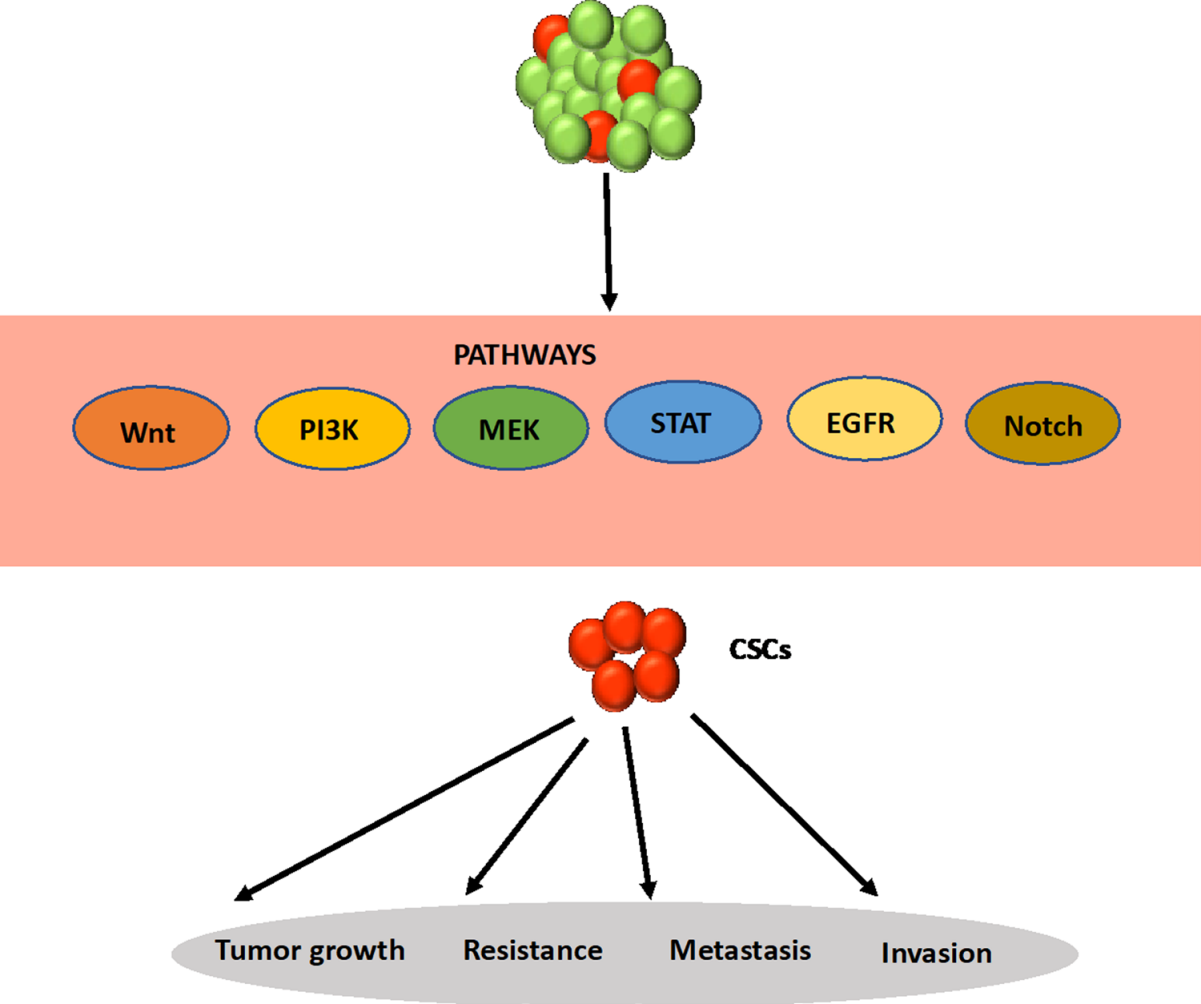
Different oncogenic signaling pathways may be active in CSCs to promote drug resistance, tumor growth, invasion, and metastasis.

Different signaling pathways may be active in CSCs (Fig. 4). Evidence supports a major role for Wnt signaling to maintain stem cell homeostasis in both normal and malignant intestine tissues [46]. In colorectal CSCs, Wnt is particularly important to maintain a stemness phenotype [47, 48]. Likewise, PI3K/Akt pathway plays a key role in CSCs biology and reprogramming of cancer cells in solid tumors [49, 50]. Moreover, in lung cancer, acquired resistance to an EGFR inhibitor led to stemness of cancer cells, suggesting a role for EGFR signaling in CSCs [51]. In addition, JAK/STAT signaling pathway is involved in maintaining embryonic stem cell self-renewal, hematopoiesis, and neurogenesis [52, 53]. It has been shown that this pathway is activated aberrantly in CSCs isolated from tumors of breast, prostate, blood, and glia. For example, stem-like cells isolated from prostate tumors overexpressed several genes in JAK/STAT signaling pathway including IFNK, IFNGR, IL6, and STAT1 [54]. Activated form of STAT3 was shown to upregulate JAK/STAT signaling pathway in breast CSCs [55]. Thus, acquired resistance of cancer cells to molecular inhibitors can result from activation of oncogenic signaling pathways and gain of cancer stemness. In summary, cancer stemness-mediated drug resistance may involve cell-intrinsic processes, such as EMT and dormancy, and cell-extrinsic factors from the tumor microenvironment, including hypoxia and specific interactions between stromal and cancer cells. From a therapy perspective, identifying such mechanisms of stemness-mediated tumor progression in the context of specific subtypes of each cancer is critical to develop new targeted therapies that leverage CSCs as a therapeutic target.

## Engineered Three-Dimensional Models to Study CSCs

Traditionally, cancer drug resistance has been studied with monolayer (2D) cell cultures. However, tumor cells in the human body reside in 3D environments that control cell behavior and fate. The architecture and mechanical properties of tumors, interactions of cancer cells with stromal cells and the extracellular matrix, and signaling of cancer cells with soluble factors in tumors are key drivers of how cancer cells respond to or adapt to treatments, evolve, and eventually develop incurable metastases [56]. Animal models used in cancer research to investigate drug responses and resistance of tumors are truly physiological models and offer many advantages over in vitro models. Nevertheless, certain limitations including the difficulty of longitudinal studies and the number of animals needed for time-point experiments [57], interspecies differences that are not translatable from animals to humans [58], lack of human stroma in animals, and cost of animal studies are major deterring factors. Thanks to investments mainly from different federal funding agencies in the two past decades, various 3D tumor models have emerged to address the need to recreate important physiological and biological aspects of human tumors. These models reproduce the morphology of solid tumors, enable addition of human stromal cells and defined extracellular matrices, and mimic key behaviors of cancer cells including drug resistance [59–61]. Below, we summarize the different types of 3D models that have been used to study drug resistance due to CSCs and to test therapeutics.

## Spheroid Model

**Fig. 5:**
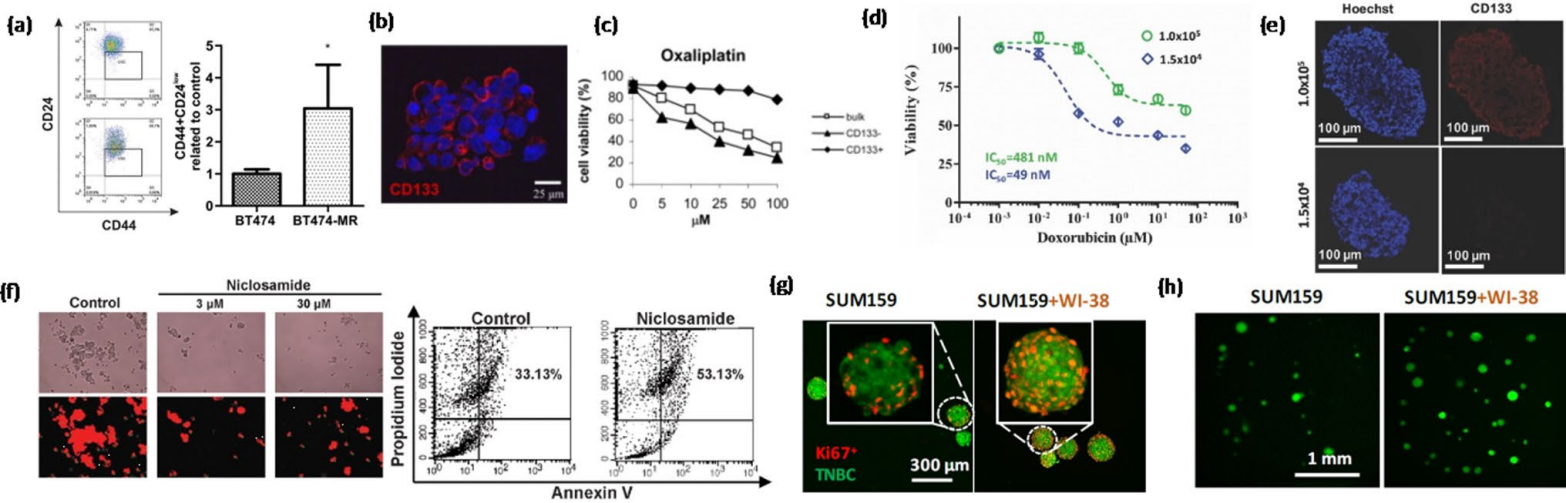
**a** Flow cytometry plots of CD24 and CD44 expression of BT474 breast cancer cells and HER2 inhibitor-resistant BT474-MR breast cancer cells ( © 2017 Wiley Periodicals, Inc). **b** Confocal microscopy image of CD133 expression on a colorectal cancer spheroid. Nuclei was stained with Hoechst (blue). **c** Cell viability following 24 h exposure of bulk, CD133^-^ and CD133^+^ cells to different concentrations of oxaliplatin (© 2007 Elsevier Inc. All rights reserved). **d** Dose-response of breast cancer spheroids to doxorubicin treatments shows drug resistance in denser, hypoxic spheroids. **e** Cryosections of breast cancer spheroids immunostained with CSC marker CD133 (red). Blue represents nuclei staining with Hoechst. (© 2016 WILEY-VCH Verlag GmbH & Co. KGaA, Weinheim) **f** Niclosamide inhibited sphere formation and decreased the fraction of spheroid forming cells in breast cancer (© 2013 Wang et al). **g** WI-38 lung fibroblast cells promoted formation of TNBC SUM159 cell colonies through HGF-Met signaling. **h** Immunofluorescence staining of Ki67 (red) in SUM159 colonies with or without WI-38 fibroblast cells. Green cells in the images are TNBC cells. (© 2022, American Association for Cancer Research). Reproduced with permission.

Spheroids are 3D compact aggregates of cancer cells grown on low-adherent tissue culture plates. Spheroids represent avascular or poorly vascularized tumors and mimic solid tumors in terms of close cell-cell contacts, spatial gradients of nutrients and oxygen, drug transport limitations, and tumor-stromal interactions when stromal cells are included in the model. Cancer cell spheroids have been successfully used to study tumor growth, proliferation, and invasion [62], micro-metastasis [63], immune-cancer cells interactions [59], and to screen cancer drugs [64]. The main scaffold-free spheroid formation techniques are based on using a spinner flask, a liquid overlay microplate, hanging drop arrays, non-adherent surface culture, and aqueous-two phase systems [65]. Hanging drop technique confines cancer cells into a pendant drop of media to induce cell aggregation into a spheroid due to the force of gravity [66]. Breast cancer cell spheroids made with the hanging drop method were enriched in CSCs and showed increased levels of pluripotent genes under treatment with a HER2 inhibitor. The resistant cells had a significantly greater CD44^+^/CD24^-^ subpopulation than the parental cancer cells (Fig. 5a) [67]. Spinner flasks and NASA rotary cell culture systems have also been used to develop spheroids that were enriched with CSCs [68]. Spheroids have been used to study therapeutic strategies against CSCs in head and neck cancer [69]. Implanting ALDH^+^/CD44^+^ CSCs in mice led to tumor formation due to signaling with endothelial cells and formed greater colonies. CSCs showed perivascular localization and selective ablation of blood vessels significantly reduced the fraction of ALDH^+^/CD44^+^ CSCs. A study of tumorigenic potential of patient-derived colon carcinoma cells also showed that CD133^+^ CSCs were inherently capable of spheroid formation (Fig. 5b) and resisted cell death under treatments with oxaliplatin (Fig. 5c) and 5-flurouracil through autocrine IL-4 production and signaling [70]. A similar finding was reported in liver cancer where CSCs showed significantly enhanced spheroid formation and chemoresistance to gemcitabine [71].

Hypoxia results from inadequate oxygen supply in tumors due to their abnormal vasculature. Hypoxia has been associated with drug resistance in various cancers [72]. Hypoxic cells activate signaling pathways such as Notch and express transcription factors such as Oct4 that control self-renewal and multipotency of CSCs [73]. Due to their spherical shape, tumor spheroids contain gradients of oxygen and nutrients, which result in a necrotic and quiescent core and a more proliferative periphery. As such, spheroids are an inherently proper model to study hypoxia-mediated changes in cancer cell functions. For example, it was demonstrated that hypoxic triple negative breast cancer spheroids made with an aqueous two-phase system showed significant resistance to doxorubicin (Fig. 5d) and cisplatin treatments and high expression of CD133 compared to spheroids without hypoxia (Fig. 5e) [74]. Similarly, breast tumor spheroids with high numbers of CD133^+^ cells had a hypoxic core and elevated HIF1α expression [75].

Tumor spheroids have also been used to study the feasibility of targeting CSCs. For example, rapamycin, an FDA-approved mammalian target of rapamycin inhibitor (mTOR), reduced the viability of CD133^+^ cells in pancreatic cancer spheroids [76]. In a different study, it was shown that resistance of BRAF^mut^ and KRAS^mut^ colorectal cancer spheroids to MEK inhibitors could be overcome by targeting CSCs using mithramycin [77]. Another study showed that breast cancer cells with high ALDH activity or high CD44 with low CD24 expressed high levels of type I tyrosine kinase like orphan receptor ROR1 and had greater capacity to form spheroids. This study demonstrated that using an inhibitor of ROR1 blocked cancer stemness and reduced the capacity to develop metastases in immunodeficient mice [78]. Breast cancer spheroids enriched with CD44^+^/CD24^-^ cells grew tumors and targeting these CSCs with niclosamide inhibited spheroid formation, induced apoptosis, and inhibited tumor growth in mice (Fig. 5f) [79]. In ovarian cancer, targeting CSCs using salinomycin reduced expression of stemness markers and spheroid forming ability of ovarian CSCs. The ovarian CSCs resisted paclitaxel treatment but its combination with salinomycin promoted apoptosis of CSCs [80].

To explore the role of tumor microenvironment on drug resistance and cancer stemness, several studies have used co-culture spheroids of cancer and stromal cells. Incorporating stromal cells in spheroids promoted oncogenic signaling in colon cancer cells [81]. Interactions of colon cancer cells with stromal cells activated Wnt/ß-catenin pathway in cancer cells and enriched CSCs [82]. Spheroids of different breast cancer cell lines and their respective stromal cells such as fibroblasts were used to study stromal cell-mediated resistance to molecular inhibitors including those of MAPK and PI3K/Akt pathways [83]. In a different study, co-culture of colon cancer cells and fibroblasts were used to test effects of cytotoxic chemotherapy drugs such as 5-fluorouracil, and targeted therapies such as erlotinib and regorafenib on colon cancer cells [84]. Pancreatic ductal adenomacarcinoma spheroids in the presence of conditioned medium from cancer-associated fibroblasts (CAFs) had elevated levels of ALDH^+^/CD44^+^ CSCs. Signaling from CAFs increased protein levels of CSC markers CD44, Nanog, ALDH1/2, and SOX9, and promoted clonogenicity and EMT phenotypes of cancer cells [85]. More recently, an organotypic tumor model comprising of a spheroid of triple negative breast cancer cells embedded in collagen containing dispersed patient-derived CAFs was developed to study tumor-stromal interactions. It was shown that CAFs predominantly secreted HGF to promote Met phosphorylation in cancer cells. This signaling activated several kinase pathways, conferring resistance to inhibitors of MAPK pathway in a long-term culture. Triple negative breast cancer cells in presence of lung fibroblasts had significantly greater proliferation and colony formation (Fig. 5g, h), indicating gain of stemness due to interactions with fibroblasts [86]. Heterotypic spheroids formed with 20% macrophages and 80% ovarian cancer cells or isolated ovarian CSCs had elevated levels of the immunosuppressive cytokine IL-10. Likewise, an increase in ALDH expression was observed in the co-culture spheroids, indicating the role of interactions with macrophages in CSC maintenance and resistance to carboplatin treatment [87].

As a widely-used 3D culture, spheroids offer a suitable model for high-throughput drug testing and screening studies due to their ease of formation and cost-effectiveness. It should be taken in to account that spheroids can develop a hypoxic and necrotic core in long-term cultures [62], impacting both oncogenic signaling activities and promoting and enriching CSCs. In addition, spheroids help replicate plasticity of CSCs, which is a known mechanism of drug resistance. That is, in the absence of treatment pressure, CSCs shift toward a proliferative state and revert to a quiescent stem cell state under drug treatment, allowing survival during chemotherapy. For example, we recently used colorectal cancer cell spheroids in cyclic treatment and recovery with molecular inhibitors to mimic how patients receive chemotherapy. We found that levels of several CSC markers were significantly higher during treatment phases but often reduced during recovery phases [77], suggesting adaptive behavior of cancer cells that display a stem cell-like behavior to escape drug toxicity. Techniques such as colony forming assay, flow cytometry, and sequencing can be leveraged to further study this mechanism in spheroid cultures [88, 89].

## Organoid Model

**Fig. 6:**
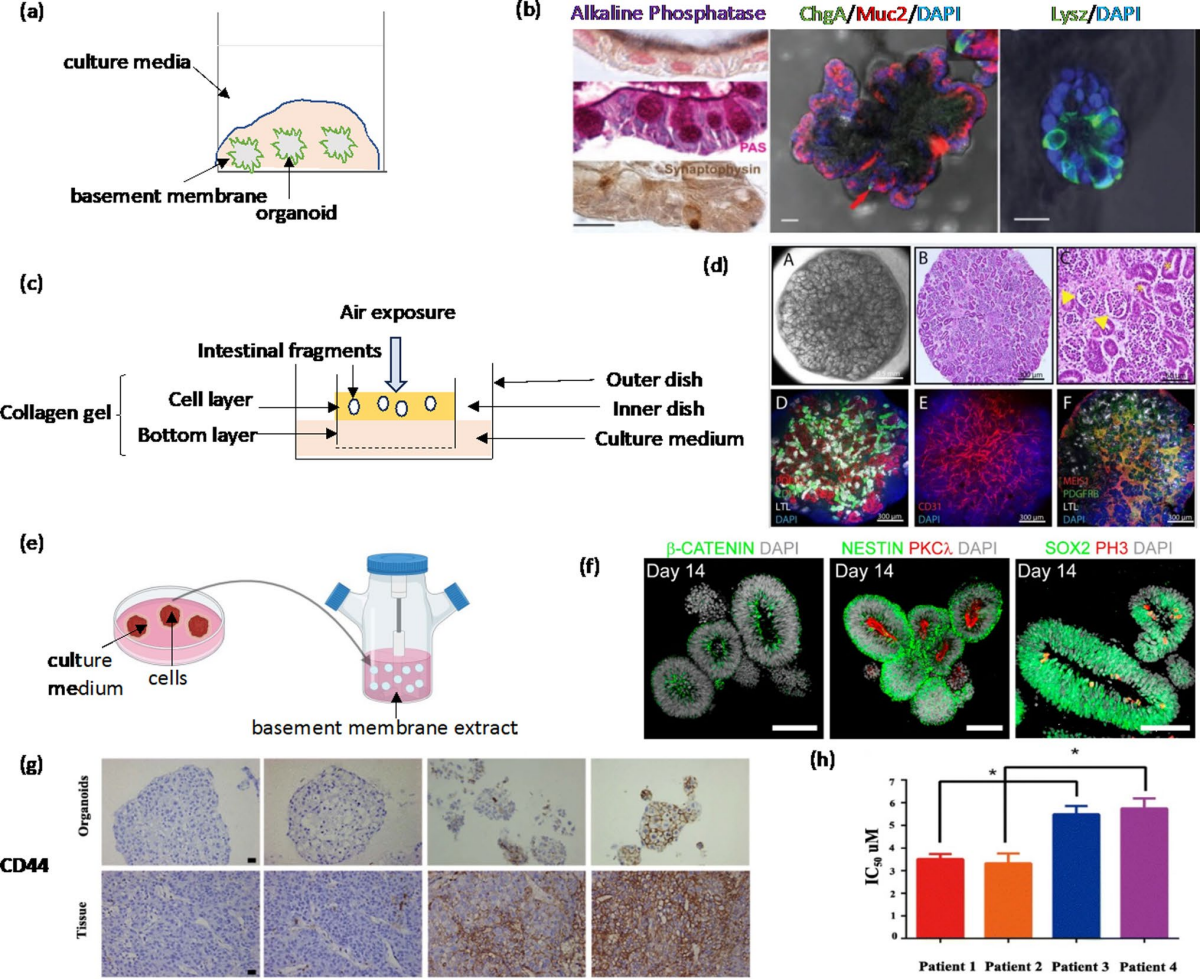
**a** Organoids result from embedding a single-cell suspension in basement membrane extract submerged in specific culture media. **b** Human intestinal organoids show alkaline phosphatase expression (purple) and Muc2 (red) for goblet cells, and ChgA and Lysz (green) for enteroendocrine and Paneth cells. (© 2011 AGA Institute, Published by Elsevier Inc.) **c** Air-liquid interface for intestinal organoid culture. **d** H & E staining of densely packed kidney organoids shows tubular structures with lumens (yellow stars). (© 2013-2021 The Journal of Biological Methods) **e** Tissue fragments were embedded in basement membrane extract followed by transfer into a bioreactor. **f** Forebrain organoids on days 14 immunostained for β-catenin, Nestin, SOX2 (green), PKCλ, PH3 (red), and DAPI (grey) (© 2016, Elsevier Inc). **g** Expression of CSC marker CD44 in hepatocarcinoma PDOs and the original tumor. **h** The IC50 values of Sorafenib were significantly higher in CD44-positive PDOs (patients 3 & 4) than in CD44-negative PDOs (patients 1 & 2) (© 2020, Siqi Wang et al). Reproduced with permission

Organoids are self-organizing, 3D microscopic structures that are derived from individual stem cells in vitro and recapitulate certain cellular and biological aspects of their respective tissues [90]. In recent years, there have been major advances in organoid technologies. Organoids may develop from adult stem cells and pluripotent stem cells [91]. For example, intestinal adult stem cells were used to produce intestinal organoids with crypt-like and villi-like regions, which resembled the spatial arrangement of these structures in vivo [92], and human pluripotent stem cells were used to form cerebral organoids and understand neurogenesis [93]. A major advance resulted from a pioneering study by Sato and Clevers, where human Lgr5-enriched intestinal stem cells were embedded in a laminin-rich basement membrane extract and supplemented with various soluble factors (Fig. 6a) [94]. Cells formed self-organizing structures resembling crypt-villus units (Fig. 6b) [95]. To mimic intestinal stem cells niche, a different type of organoid culture was developed using a collagen gel-based air-liquid interface (ALI) in cell culture inserts known as Boyden chambers. This approach allowed forming organoids of small and large intestines [96]. Cells were embedded in an extracellular matrix hydrogel in the upper compartment, which was exposed to air. The bottom compartment contained collagen hydrogel. Culture medium placed in the outer dish diffused through the collagen hydrogel and across the porous membrane between the two compartments to nourish the cells (Fig. 6c) [97]. The ALI culture allowed the propagation of kidney organoids that were densely packed with tubular clusters and tubules containing lumens (Fig. 6d) [98]. In another approach, brain cells were embedded in drops of the basement membrane and then transferred into spinning bioreactors (Fig. 6e) to produce human brain region-specific organoids [93]. Organoids showed neural-tube like structures, were positive for adherent junction markers, and were proliferative near the ventricular surface (Fig. 6f) [99].

Organoids can also develop from tumor cells with stemness properties. Cells harvested from an individual patient’s tumor biopsy are embedded in a hydrogel to create tumor organoids that mimic cell heterogeneity and signaling of the respective parental tumor [100]. Such patient-derived organoids (PDOs) have been used to study initiation, progression, and invasion of cancer cells and hold a great promise to develop patient-specific treatments toward realizing personalized cancer medicine. Like spheroid models, tumor organoids can be used to study drug responses of cancer cells. Several studies have established that PDOs closely replicate drug responses both in mice and in human patients [101–103]. For example, PDOs were used to evaluate patients’ responses and resistance to drugs [102, 104]. PDOs were generated from 110 biopsies of 71 patients with metastatic, heavily pretreated colorectal and gastroesophageal cancers. PDOs showed morphological similarities to their parental tumor tissues. Paclitaxel responses of PDOs from a paclitaxel-sensitive patient and two patients with liver metastases that were resistant paclitaxel were evaluated. Results showed increased viability in patients with primary or acquired resistance to paclitaxel [102].

Organoids and a PDO-based orthotopic mouse tumor xenografts accurately predicted drug response and resistance in metastatic gastric cancer. In this study, PDO-xenografts from a gastrointestinal cancer patient with primary resistance and a patient responding to a multi-kinase inhibitor, regorafenib, were used [102]. The PDO-xenografts from regorafenib-sensitive patient showed reduction in micro-vasculature, which was consistent with clinical findings in regorafenib-treated gastrointestinal tumors [105]. In a separate study, PDOs formed with four different hepatocarcinoma tumor specimens were treated with a multi-kinase inhibitor, sorafenib. CSC containing CD44^+^ organoids were significantly less sensitive to sorafenib than CD44^-^ organoids (Fig. 6g, h). Sorafenib treatment upregulated CD44 but a Hedgehog signaling inhibitor, GANT61, decreased cell proliferation and increased apoptosis in the hepatocarcinoma organoids and suppressed CSCs. A combination of sorafenib and GANT61 effectively decreased colony size and cell viability, especially in CD44^+^ PDOs [106].

PDOs generated from colorectal tumor specimens showed resistance to monotherapies with 5-Flurouracil and Irinotecan, whereas the combination of each drug with Hedgehog inhibitors significantly inhibited the expression of c-Myc, CD44, and Nanog, and improved drug responses [107], highlighting the potential to eradicate colorectal CSCs by targeting Hedgehog pathway. Because PDOs enable predicting drug responses and conducting mechanistic studies, several PDO biobanks have been created, such as for metastatic breast cancer [108], and for breast cancer patient-derived xenografts and matched PDOs [109]. The patient-derived xenograft organoids showed similar phenotypic and genotypic characteristics to their parental tumors, such expression of CSC markers ALDH1A3, CD133, and CD44. The organoids effectively emulated treatment responses in a 43-year-old individual diagnosed with stage IIA triple negative breast cancer. Specifically, the patient received a treatment regimen involving doxorubicin and cyclophosphamide followed by paclitaxel (AC-T therapy) and surgical pathology, which resulted in complete remission. Moreover, PDOs from tumors resistant to AC-T therapy were sensitive to an alternative drug, eribulin. This treatment resulted in remission for an additional two months, mirroring the outcome experienced by the patient undergoing this alternative treatment [109]. Due to accurately reflecting patient drug responses, PDOs provide a model of practical significance and can potentially guide clinicians in selecting effective treatment strategies for cancer patients. Organoids are a valuable tool for both mechanistic studies in academic and industrial research laboratories and for evaluating patient-specific drug responses in clinical laboratories. A main limitation of organoids is their heterogeneity in terms of size and cellular composition, which can affect reproducibility and interpretation of results, and the need for mainly animal-derived matrices to form them [110].

## Microfluidic Model

**Fig. 7:**
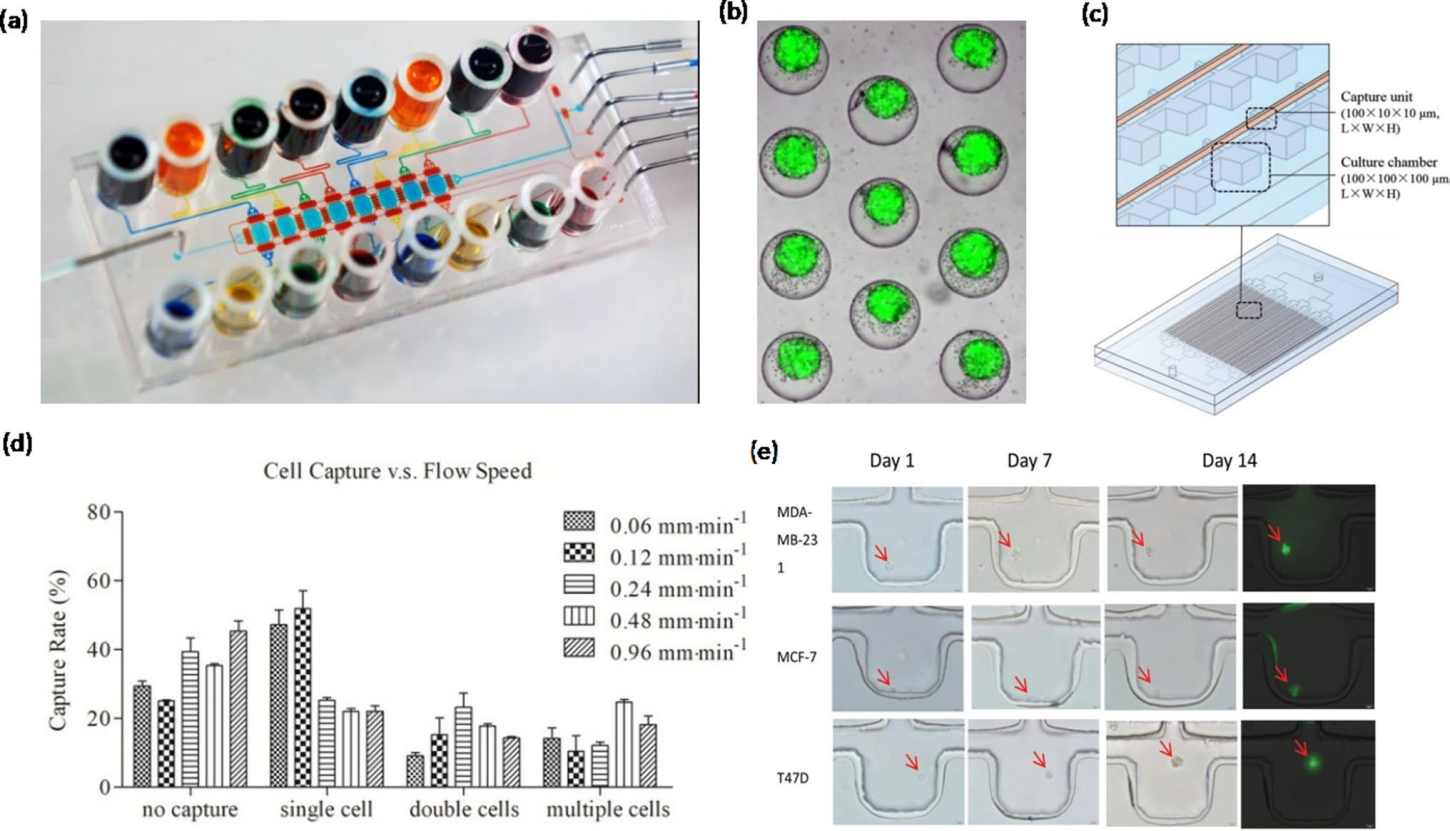
** a** A microfluidic device configured to serially connect culture chambers. **b** Spheroids formed after seeding cells in the device (© 2020, Neda Daggar et al.). **c** Single cell-array microfluidic chip and the cell capturing unit. **d** Cell-capturing rates in the microfluidic device at different flow rates. **e** Clone formation of MDA-MB-231, MCF-7, and T47D cells (© 2019 WILEY-VCH Verlag GmbH & Co. KGaA, Weinhelm). Reproduced with permission.

Tissue-engineered microfluidic devices leverage engineering principles to control the microenvironment of cells. Sophisticated microfluidic devices have been developed to replicate tissue- and organ-level functions in vitro [111]. Microfluidic organ-on-chip models have been established for a variety of organs, including lung [112, 113], liver [114], kidney [115], and heart [116]. Recently, a microfluidic device was used to isolate and characterize CSCs from pancreatic ductal adenocarcinoma patients. It was found that samples were positive for CSCs (CD133^+^/CK^+^) for stage IV, pancreatic ductal patients. An increase in the number of CSCs was seen in specimens with progression of cancer [117], suggesting a potential role for CSCs in disease progression. Several microfluidic devices have also integrated spheroid cultures for high throughput screening of cancer drugs [118, 119]. For example, primary ovarian tumor cells were used in a microfluidic device with integrated microvalves to generate spheroids of different cell densities and for serial drug dilution to facilitate dose-response studies (Fig. 7a, b) [120]. Microfluidic devices have also proved useful to screen for CSC biomarkers. For example, a device with 3840 cell capturing units was used to identify breast CSCs (Fig. 7c) [121]. This study demonstrated a single cell capturing efficiency of ~ 60% (Fig. 7) and evaluated clone forming capacity of single cells, i.e., a key property of CSCs. MCF-7, MDA-MB-231, and T47D cells respectively showed 1.67%, 5.78%, and 5.24% clonogenic capability (Fig. 7e). This device also allowed testing the efficacy of several drug compounds such as paclitaxel, doxorubicin, thiostrepton, and salinomycin against breast cancer cells. Microfluidic devices also allow studies of tumor-stromal interactions. For example, 3D co-culture models of liver CSCs and endothelial cells or hepatocarcinoma cells and endothelial cells were developed in Matrigel within a microfluidic device. CSCs induced endothelial cell migration more efficiently than hepatocarcinoma cells did. Endothelial cells also significantly enhanced invasiveness of CSCs than hepatocarcinoma cells did, indicating the role of bi-directional intercellular interactions on dynamic remodeling of the tumor microenvironment and the resulting local invasion of CSCs [122]. Among in vitro models, microfluidic devices offer the most control over the microenvironment of cells and reproducing specific processes during tumor progression. While microfluidic devices are suitable for small-scale phenotypic studies, conducting mechanistic studies or using them for large-scale applications such as drug screening is challenging due to difficulties with fabricating and packaging them at a large scale, maintaining cell cultures within these devices beyond a few days, and need for specialized equipment to facilitate dynamic cultures.

## Challenges and Future Directions

3D tumor models offer a significant potential to replicate physiologically relevant tumor environments. Organoids and spheroids can be generated from tumor biopsies of patients to test and identify effective treatments for each individual patient. Availability of molecular profiling of tumors facilitates this personalized cancer treatment approach to guide treatment decisions [109]. A major benefit of these models is the ease of generating organoids and spheroids in high throughput to screen various compounds as monotherapies or combination therapies. Research studies with PDOs that were highlighted above indicate the feasibility of this approach.

3D tumor models can be used in long term cyclic treatment/recovery to mimic how patients receive chemotherapies and determine changes in drug responses of cancer cells over time. For example, colorectal cancer spheroids showed increased resistance to a specific protein kinase inhibitor, trametinib, elevated expression of CSC markers, and an increased clone formation capacity during treatment phases [77]. Interestingly, removal of the drug during recovery phases reduced cancer stemness, indicating the utility of the model to capture the plasticity of CSCs that revert from a proliferative state to a quiescent state under drug pressure and gain a proliferative state in the absence of drug pressure. Thus, 3D tumor models facilitate the understanding of cell-intrinsic and extrinsic drug resistance mechanisms to target such mechanisms toward developing treatment strategies with sustained effectiveness against cancer cells.

The tumor microenvironment plays a key role in promoting stemness of cancer cells and their drug resistance. Interactions of stromal and immune cells with cancer cells through different mechanisms, e.g., soluble signaling and direct contacts, can cause drug resistance [123–125] . Future studies should incorporate stromal cells and immune cells in engineered tumor models and unravel specific mechanisms by which these interactions promote cancer stemness and identify effective approaches to target them. Paired normal and tumor organoids containing stromal cells, all derived from the same patient, could be used for such mechanistic studies to test and offer treatment regimens for the individual.

A major limitation in organoid technology has been using animal-derived basement membrane extracts, such as Matrigel and Cultrex, which cause variabilities and inconsistencies in resulting organoids and fail to mimic the extracellular matrix of the tumor environment. Developing new formulations of matrices that are fully human-derived is a critical future development. Synthetic hydrogels with highly tunable biological and mechanical properties provide an alternative solution [126].
